# The Potential of Web-based Interventions for Heart Disease Self-Management: A Mixed Methods Investigation

**DOI:** 10.2196/jmir.1438

**Published:** 2010-12-02

**Authors:** Cicely Kerr, Elizabeth Murray, Lorraine Noble, Richard Morris, Christian Bottomley, Fiona Stevenson, David Patterson, Richard Peacock, Indra Turner, Keith Jackson, Irwin Nazareth

**Affiliations:** ^8^British Cardiac Patient AssociationNottinghamUnited Kingdom; ^7^Unaffiliated patient representativeLondonUnited Kingdom; ^6^Archway Healthcare LibraryLondonUnited Kingdom; ^5^The Clinical and Academic Department of Cardiovascular MedicineWhittington Hospital NHS TrustLondonUnited Kingdom; ^4^Department of Infectious Diseases and EpidemiologyLondon School of Hygiene & Tropical MedicineLondonUnited Kingdom; ^3^Department of Primary Care and Population HealthUniversity College LondonLondonUnited Kingdom; ^2^Academic Centre of Medical EducationUniversity College LondonLondonUnited Kingdom; ^1^E-Health UnitDepartment of Primary Care and Population HealthUniversity College LondonLondonUnited Kingdom

**Keywords:** Internet, Coronary disease, Heart diseases, Primary health care, Self care, Selective enrolment, Digital divide, Healthcare disparities

## Abstract

**Background:**

Existing initiatives to support patient self-management of heart disease do not appear to be reaching patients most in need. Providing self-management programs over the Internet (web-based interventions) might help reduce health disparities by reaching a greater number of patients. However, it is unclear whether they can achieve this goal and whether their effectiveness might be limited by the digital divide.

**Objective:**

To explore the effectiveness of a web-based intervention in decreasing inequalities in access to self-management support in patients with coronary heart disease (CHD).

**Methods:**

Quantitative and qualitative methods were used to explore use made of a web-based intervention over a period of 9 months. Patients with CHD, with or without home Internet access or previous experience using the Internet, were recruited from primary care centers in diverse socioeconomic and ethnic areas of North London, UK. Patients without home Internet were supported in using the intervention at public Internet services.

**Results:**

Only 10.6% of eligible patients chose to participate (N=168). Participants were predominantly Caucasian well-educated men, with greater proportions of male and younger CHD patients among participants than were registered at participating primary care practices. Most had been diagnosed with CHD a number of years prior to the study. Relatively few had been newly diagnosed or had experienced a cardiac event in the previous 5 years. Most had home Internet access and prior experience using the Internet. A greater use of the intervention was observed in older participants (for each 5-year age increase, OR 1.25 for no, low or high intervention use, 95% CI, 1.06-1.47) and in those that had home Internet access and prior Internet experience (OR 3.74, 95% CI, 1.52-9.22). Less use was observed in participants that had not recently experienced a cardiac event or diagnosis (≥ 5 years since cardiac event or diagnosis; OR 0.69, 95% CI, 0.50-0.95). Gender and level of education were not statistically related to level of use of the intervention. Data suggest that a recent cardiac event or diagnosis increased the need for information and advice in participants. However, participants that had been diagnosed several years ago showed little need for information and support. The inconvenience of public Internet access was a barrier for participants without home Internet access. The use of the intervention by participants with little or no Internet experience was limited by a lack of confidence with computers and discomfort with asking for assistance. It was also influenced by the level of participant need for information and by their perception of the intervention.

**Conclusions:**

The availability of a web-based intervention, with support for use at home or through public Internet services, did not result in a large number or all types of patients with CHD using the intervention for self-management support. The effectiveness of web-based interventions for patients with chronic diseases remains a significant challenge.

## Introduction

Support for patient self-management is central to healthcare strategies for managing patients with chronic diseases [1,2]. For patients with heart disease, self-management education is usually provided as a component of a cardiac rehabilitation program [3] or through more generic chronic disease initiatives such as the Chronic Disease Self-Management Program (CDSMP) in the USA [4] and the Expert Patients Programme (EPP) in the UK [5]. However, low enrolment is a problem for these programs, and concerns have been raised over whether they are reaching those most in need [6,7]. For example, fewer than 30% of eligible patients enroll in cardiac rehabilitation programs [6] and initial evaluations of the EPP found that 75% of programs experienced recruitment difficulties [8] and enrolled predominantly highly educated participants [8-10]. The CDSMP and EPP programs have a predominance of Caucasian and female participants [8,10], whereas cardiac rehabilitation programs have a disproportionately high number of younger male participants [9].

Reducing healthcare disparities is a major health policy goal in many countries [11,12]. It has been suggested that delivering self-management interventions over the Internet (web-based interventions) may reduce disparities in access to these programs by overcoming many of the practical barriers that hinder attendance to programs that use a one-on-one approach [13,14]. Web-based interventions also have the potential to overcome educational barriers by presenting complex information in a more easily accessible manner, for example, through animations or video. Systematic review evidence suggests that web-based interventions can achieve health benefits in patients with chronic diseases [15], and qualitative research suggests that patients see the potential of web-based interventions for meeting their information and support needs [16].

However, while access to the Internet increases on a yearly basis; it is not equally accessible [17-22]. Although 70% of the general population in the UK had home Internet access in 2009 [17,19], access was much lower in less advantaged populations: 38% among those with the lowest annual income (< £12,500 per year, equivalent to US$ 20,000, €13,800), and 49% among those with only basic education [19]. Relatively low Internet use (41%) has also been found among people with health problems or disabilities [19], in older individuals (30% of those ≥65), and among women [17]. Similar disparities exist in the US [12,16], Canada, and other countries [17].

Despite the relative lack of access to the Internet amongst disadvantaged groups, individuals in these groups seem to make relatively high use of the Internet for their health information needs. Women, and individuals with chronic diseases in particular, use the Internet to obtain health information [23-25]. Those in poorer health and in lower income brackets are more likely to use health-focused online support groups [23]. Individuals with chronic diseases and those in older age groups use the Internet for social networking and for obtaining health information as much as those without health problems and those in younger age groups, respectively [24,25]. Increasing use of the Internet for obtaining health information has been observed in patients with heart disease [26].

As a result, there is uncertainty as to whether the lack of equity in Internet access (the digital divide) results in increased health disparities [27-28], or whether other factors such as enhanced comprehension and greater use by relevant groups can increase the equity in the use of web-based self-management programs.

To date, most evaluations of web-based interventions for patient self-management have been limited to patients that already have Internet access. Studies that attempted to be more inclusive provided computers and home Internet access to participants for the duration of their studies [29,30]. They showed increased benefits to participants and, as a result, provide further support for the potential value of these types of interventions in patients previously without Internet access. However, this approach is costly and unlikely feasible outside of a research setting.

An alternative approach is to encourage access to web-based interventions at public Internet facilities. This is possible in the UK due to government investment in the provision of free public Internet access, support, and training aimed specifically at overcoming the digital divide [31]. However, whether public Internet access facilitates the use of online self-management support by individuals with chronic diseases remains unclear.

The objective of this study was to explore the potential of a web-based intervention for reaching a large number of patients, including those in disadvantaged groups, by examining: (1) the participation level in a study evaluating a web-based intervention for coronary heart disease (CHD), and (2) the level of use of the intervention by the participants. The study aimed to be inclusive by recruiting participants from primary care centers that offer services to diverse ethnical and socioeconomic communities, and by providing support to patients that had no prior Internet experience or home Internet access.

## Methods

### Design

This prospective cohort study examined the level of use of a web-based intervention by primary care patients with CHD over a 9-month period. The study used both quantitative and qualitative methods. The methods were designed to complement each other by examining the topic from two perspectives: the statistical investigation of the level of use of the intervention and exploration of individual patient experiences of the intervention. Ethics and research governance approval were obtained from the Camden and Islington Local Research Ethics Committee and the appropriate primary care trusts.

### Recruitment

General practices in the UK maintain accurate and up-to-date registers of patients with long-term conditions, including CHD. One hundred sixty-eight (N=168) patients on the CHD registers of 10 primary care centers in North London, UK, were recruited for this study. The centers were selected based on the diversity of the communities they serve and the research interests of their general practitioners (GPs). All centers served populations that ranked in the most deprived quintile of the UK population, based on Townsend deprivation scores [32]. These scores are a summary measure of relative material deprivation within small populations based on 4 indicators from Census data: unemployment, overcrowding, lack of owner occupied accommodation, and lack of car ownership. Positive scores indicate a higher rate of material deprivation and negative scores represent the opposite [33]. Recruitment was as inclusive as possible and based on the following criteria. Inclusion criteria included patients with a diagnosis of CHD registered at a participating North London general practice, and patients who were willing to visit a local public Internet service or had Internet access at home. Exclusion from the study were: patients who were terminally ill (< 9-month life expectancy); patients unable to provide informed consent due to mental impairment; patients unable to speak English well enough to consult without an interpreter; and patients unable to use a computer due to visual, hearing, or motor impairment.

Physicians at the participating centers screened patients from the CHD register and excluded patients based on the exclusion criteria. Eligible CHD patients were sent a written invitation to participate in the study. Recruitment materials specified that participants with no previous computer or Internet experience and/or without home Internet access were welcome to participate. Housebound patients with home Internet access were included but those without were excluded.

### Web-based Intervention

**Figure 1:**
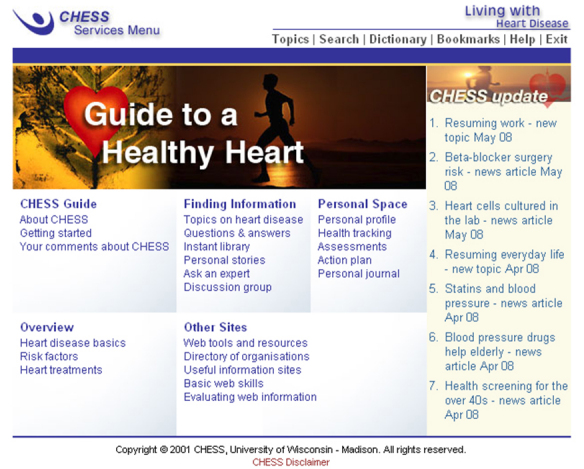
Screen shot of the home page (services menu) of the CHESS Living with Heart Disease web-based intervention used in this study.

The Comprehensive Health Enhancement and Social Support (CHESS) Living with Heart Disease web-based intervention used in this study provided interactive information, behavior change support, and peer and expert support components. It was designed by the CHESS Team at the University of Wisconsin [34] and was further developed for this study [35]. Figure 1 shows a screenshot of the final intervention.

To help overcome the digital divide, participants received individual training in how to use the intervention and were provided information on local, free, or low-cost public Internet services and training courses. Training was tailored to each participant’s level of Internet experience. Training of patients without home Internet access was conducted at a local public Internet service (eg, library, Internet café, community centre). This included a booklet for each participant to record login details, contact details for assistance, a summary of the intervention services, and details of local Internet services and courses. Participants were encouraged to contact the research team for further training when necessary and were offered further training if they had not used the intervention within a month of initial training.

### Data Collection

#### Quantitative Data

Participants completed a questionnaire that provided demographic details, CHD history, and information about their Internet experience and accessibility to the Internet. Clinical information was cross-referenced with GP records for participants that consented (N=160, 95%). Consent to this aspect of the study was optional due to ethical requirements. Participants also completed standard validated questionnaires including illness perception [36], perceived social support [37], and emotional status [38]. The intervention was programmed to automatically record frequency of logins and pages viewed by the individual users. Based on this data, overall level of intervention use and use of different intervention components were calculated for each participant.

The 10 participating GP practices provided limited demographic summary data from their CHD registers that allowed a limited comparison between the study sample and the general CHD population. Data and reports from UK population surveys were used to evaluate the representativeness of the study sample, based on level of education and level of Internet access [17,19,39].

#### Participant Interviews

Individual semi-structured interviews, typically lasting 20 to 40 minutes, were conducted with a subsample of participants (n=19). Each participant was given the opportunity to volunteer for interview in a questionnaire completed at the end of the 9-month period of Internet access to the intervention. Participants with a range of demographic characteristics, prior Internet experience, and level of use of the intervention were selected for interview. Characteristics of the subsample of participants who were interviewed are shown in Table 1. Sampling continued until no new themes emerged from interviews.

Interviews consisted of general and follow-up questions that were developed following discussion with clinicians, a medical sociologist, and user representatives, with the intent of exploring each participant’s perceptions, level of use of the intervention, and personal experience of the intervention. Discussion of factors influencing the use of the intervention was also initiated by more focused questions about whether participants had used the intervention as much as they expected to, when they were most likely to use it, and when had they found it useful or helpful.

Interviews were conducted in person by one researcher (CK) and recorded. Brief notes were made after each interview to record contextual information. Interviews were conducted in small batches of 3 to 4 at a time to allow an iterative process of data collection and analysis, as per good practice guidelines for qualitative analysis [40].

### Analysis

#### Statistical

Data on level of intervention use were highly skewed. As a result, the total number of intervention web pages viewed by each participant was converted into three categories of use (no, low, and high). No included those that made zero page requests. Those that made at least one page request were assigned to low- and high-use categories by median split. Multivariable analyses was performed using a proportional odds model to examine predictors of level of intervention use. Analysis was performed using SPSS^®^ software, version 15 (SPSS UK Ltd. Surrey, UK) [41].

To ensure sufficient power of analysis, the number of predictors selected for inclusion was limited to 10. Predictors were selected based on a priori observed correlation and statistical grounds. Age, gender, level of education, availability of home Internet access, and level of Internet experience were selected a priori because of their importance as factors in the digital divide. Availability of home Internet access and level of Internet experience were combined into one variable to avoid multicollinearity in the regression model. Clinical variables (eg, time since most recent cardiac event or diagnosis) and other predictors (perception of illness identity, depression, and perceived social support) were selected on the basis of sufficient variation in scores, correlation with intervention use, and relatively low correlation with other predictors.

#### Qualitative

Thematic analysis of interview transcripts was performed concurrently with data collection. This allowed for later interviews to define, extend, and clarify emerging themes. It also helped determine when no new themes were emerging and, as a result, additional interviews were no longer required.

Three members of the research team (CK, EM, FS) discussed the interview notes and transcripts before emerging themes were presented to a multidisciplinary project steering group for their feedback. Qualitative analysis was performed using Atlas.ti software, version 5 (Chicago, IL, USA) [42].

## Results

### Participants

#### Sample Recruitment

**Figure 2:**
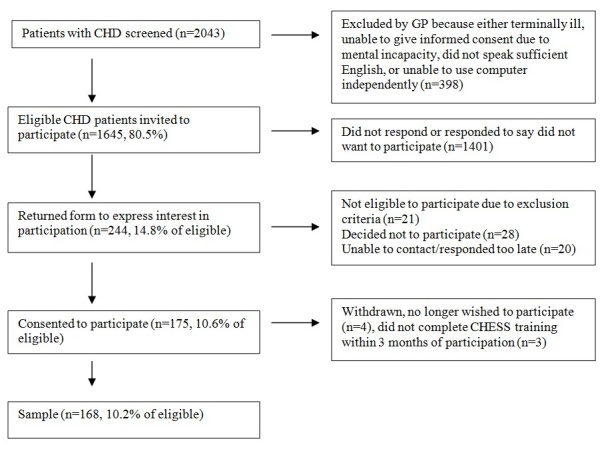
Sample recruitment

Although more than 80% of patients with CHD registered at participating centers were eligible (N=1645), only about 10% of them chose to participate (N=168), as observed in Figure 2.

#### Sample Characteristics

Patients with CHD that participated in the study were predominantly male, well educated, and Caucasian (Table 1). Close to 50% of participants had been diagnosed with CHD more than 10 years prior to the study, and very few had been diagnosed with CHD for the first time in the preceding 2 years. A greater proportion of participants had experienced a cardiac event (MI, surgical intervention, emergency hospitalization, or additional CHD diagnosis (eg, heart failure)) in the preceding 2 years. However, almost 40% of participants had not experienced a cardiac event or CHD diagnosis in the previous 5 years. Most participants had home Internet access (80%) and/or were experienced Internet users (60%) (Table 1).

**Figure 3:**
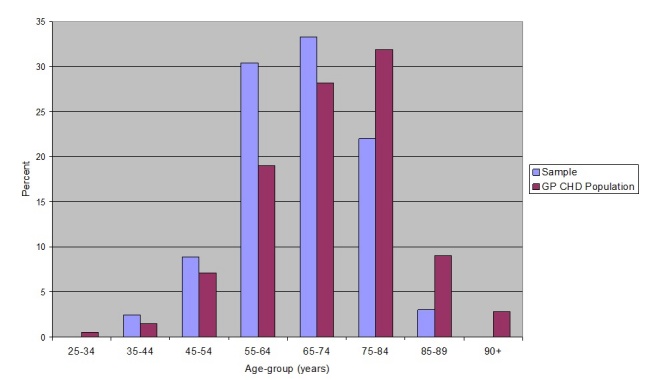
Age distributions of sample and CHD patients registered at participating practices.

Men were overrepresented in the sample, since more than 80% of participants were male compared to fewer than 65% of patients with CHD from the participating centers. The sample contained a wide spread of ages, with a mean age of 66.8 years (SD=10.1). Compared to the data for patients with CHD registered at the centers, study participants were relatively young and patients over 75 years-of-age were underrepresented (Figure 3).

Compared to UK population surveys, the number of participants with advanced levels of education, home Internet access and experience using the Internet was high. In the 2005 Health Survey for England, fewer than 8% of respondents with heart attack or angina had an advanced level of education [39] compared to 45% of participants in this study. The proportion of participants (80%) in this study that had home Internet access and/or some prior experience with using the Internet was much higher than the 41% of patients with a disability or chronic health problem that reported Internet access or Internet use in a recent population survey [19]. The proportion was also much higher than that shown in adults over 65 years-of-age that reported having used the Internet (30%) [17].

**Table 1:** Sample characteristics

		Sample (N = 168)	Interview subsample (n = 19)
Age (years)	Mean (standard deviation)	66.8 (10.1)	71.0 (8.8)
	Range	38-87	53-82
Gender	Male	137 (81.5%)	13
	Female	31 (18.5%)	6
Employment	Employed (full or part-time)	31 (18.5%)	1
	Self-employed	34 (20.2%)	1
	Full-time care	6 (3.6%)	2
	Retired	80 (47.6%)	12
	Unemployed or not working for other reasons	16 (9.5%)	3
	Not disclosed	1 (0.6%)	0
Level of education	School leaver (no further/higher qualifications)	57 (33.9%)	9
	A levels or vocational equivalent	32 (19.0%)	4
	Degree or equivalent	76 (45.2%)	6
	Not disclosed	3 (1.8%)	0
Ethnic group	White (British, Irish, other)	141 (83.9%)	14
	Black (British Caribbean, African, other)	9 (5.4%)	2
	Asian (British Indian, Pakistani, Bangladeshi, other)	14 (8.3%)	3
	Other (Chinese, other)	4 (2.4%)	0
Heart disease	Angina only	57 (33.9%)	9
	MI only	38 (22.6%)	4
	Both	46 (27.4%)	4
	Other CHD (diagnosed without angina or MI)	27 (16.1%)	2
Comorbidities	Cardiovascular comorbidity only (including diabetes, stroke)	26 (15.5%)	2
	Non-cardiovascular comorbidity only (eg, arthritis)	49 (29.1%)	3
	Both cardiovascular and other comorbidities	42 (25.0%)	8
	No comorbidity	51 (30.4%)	6
Time since earliest CHD diagnosis (years)	Mean (standard deviation)	10.6 (7.3)	9.8 (6.5)
	Range	0-35	1–22
	Diagnosed in the last year	2 (1.2%)	0
	Diagnosed 1-2 years ago	22 (13.1%)	4
	Diagnosed 3-5 years ago	28 (16.7%)	2
	Diagnosed 6-10 years ago	37 (22.0%)	3
	Diagnosed >10 years ago	77 (45.8%)	9
	Earliest CHD diagnosis given as rheumatic fever in childhood	2 (1.2%)	1
Time since most recent cardiac event (years)	Range	0-21	0–15
	Mean (standard deviation)	5.4 (4.9)	3.7 (3.6)
	Cardiac event in the last year	21 (12.5%)	1
	Most recent cardiac event 1-2 years ago	44 (26.2%)	8
	Most recent cardiac event 3-5 years ago	42 (25.0%)	7
	Most recent cardiac event 6-10 years ago	32 (19.0%)	2
	Most recent cardiac event >10 years ago	29 (17.3%)	1
Home Internet access	No	34 (20.2%)	6
	Yes	134 (79.8%)	13
Level of Internet experience	None	35 (20.8%)	6
	Basic (used a few times but not often)	32 (19.1%)	5
	Experienced or expert (regular Internet use)	101 (60.1%)	8

### Use of the Intervention

The intervention was used at least once by 77% (129/168) of the participants. However, participants varied greatly as to the frequency of using the intervention during the 9-month period (logins: range, 0-149; 10th-90th percentile, 9-23).

Median use over 9 months among participants that made at least some use of the intervention was 4 logins or viewing 148 pages of the intervention. Table 2 shows the characteristics of participants categorized as making no, low- and high-use of the intervention over 9 months (viewing 0 pages, ≤148, or >148 pages, respectively).

**Table 2:** Characteristics of participants by level of intervention use

Participant characteristics N = 168		Level of overall intervention use		
		No use: zero intervention web-pages viewed (n = 39)	Low use: ≤148 intervention web-pages viewed (n = 66)	High use: >148 intervention web-pages viewed (n = 63)
Age (years)	Mean (SD)	66.3 (9.6)	65.0 (9.7)	69 (10.6)
Gender	Male (n=137)	31 (23%)	55 (40%)	51 (37%)
	Female (n=31)	8 (26%)	11 (36%)	112 (38%)
Level of education^a^	School drop-out (n=57)	11 (19%)	22 (39%)	24 (42%)
	A levels or equivalent (n=32)	5 (16%)	16 (50%)	11 (34%)
	Degree or equivalent (n=76)	22 (29%)	27 (35.5%)	27 (35.5%)
Time since most recent cardiac event or diagnosis (years)	Mean (SD)	5.6 (4.6)	6.3 (4.9)	4.2 (5.0)
Level of Internet experience and home access	Basic or no experience, without home access (n=31)	11 (35.5%)	11 (35.5%)	9 (29%)
	Basic or no experience with home access (n=36)	6 (17%)	17 (47%)	13 (36%)
	Experienced or expert, most with home access (n=101)	22 (21%)	38 (38%)	41 (41%)

^a^ n=3, level of education not disclosed

### Factors Influencing Use of the Intervention

Proportional odds regression analyses of all complete cases of data (N=161) found that participants that were older, had more recently experienced a cardiac event or diagnosis, had home Internet access and experience using the Internet, were more likely to make some or high use of the intervention (Table 3). Gender and level of education did not predict levels of overall intervention use. Qualitative analysis confirmed the importance of several significant predictors of intervention use. Content and illustrative quotes from these themes suggest how these factors influenced intervention use and are presented below.

**Table 3:** Results of ordinal regression analyses predicting overall level of intervention use (no use, low use or high use)

Baseline predictors		Multivariable analysis	
		Odds ratio (95% confidence interval)	*P-*value
Age		1.25^a^ (1.06-1.47)	.01
Time since most recent cardiac event or diagnosis		0.69^a^ (0.50-0.95)	.03
**Internet experience and availability of home access**			
	Basic or no experience, without home access	1.00 Reference category	.01
	Basic or no experience, with home access	2.85 (1.02-7.93)		
	Experienced or expert, most with home access	3.74 (1.52-9.22)		
Perception of illness identity (symptoms experienced)		1.13 (0.99-1.29)	.07
Depression		1.06 (0.94-1.19)	.31
**Level of education**			
	School leaver	1.00 Reference category	.10
	A levels	1.40 (0.55-3.56)	
	Degree	0.61 (0.29-1.28)	
**Gender**			
	Female	1.00 Reference category	.36
	Male	1.44 (0.66-3.15)	
Perceived social support (information and emotional)		0.85 (0.62-1.18)	.33
Model Fit (compared to intercept only)		.002	

^a^ Odds ratio calculated for 5-year increase

#### Time Since Most Recent Cardiac Event or Diagnosis

The length of time since receiving a diagnosis of CHD or experiencing a cardiac event was related to participant level of need for CHD information, advice, or support. Many participants believed that they were well informed about heart disease, and this seems to have reduced their need for further assistance.

82-year-old male, experienced Internet user P0101: “I felt that I’d gone well past that stage because I’ve had my heart problem for 17 years. And as I said before, before CHESS came along I was already reasonably informed about most of the problems that would help me in my problem, how to deal with it.

They also had few questions or concerns about their disease, because they were not currently experiencing problems and generally reported feeling well and able to carry on their normal lives.

79-year-old male, basic Internet experience P0110: “I’m glad that you are doing this because it possibly could have helped me but I suppose I’m fortunate that I haven’t got a problem and therefore I didn’t need any.”

Participants experiencing recent heart disease complications reported use of the web-based intervention program to obtain new information and advice.

64-year-old male, experienced Internet user P0112: “one serious problem and one piece of information I needed to know came up as a result of my heart problems and I just, at that time, could not find the answer and CHESS… gave me the answer…it’s been very useful to tell me what was going on after my situation changed”

#### Home Internet Access

The convenience of using the web-based intervention at home was particularly appreciated.

79-year-old female, experienced Internet user P0121: “Well yes I could go up and have a look at it, you see, it was great, great just to press a couple of buttons and you’re there… I could go upstairs any time and look to see if I could find the answer up there.”

With a couple of notable exceptions, those without home Internet access reported that lack of home Internet availability was a barrier to intervention use.

81-year-old female, no previous Internet experience P0320: “… just the effort of getting out, going to the library and doing it, I know I would have done better with one [at home] because often I felt like doing that sort of in the evening… I didn’t like the forward planning, I’d have liked of just sort of get out the old computer, put it down and do it when I felt like it”

Two participants that did make high use of the intervention at local public Internet services reported having unlimited and free access to the Internet, and in one case, extensive technical support from staff. They reported added benefits to accessing the intervention away from home, such as getting uninterrupted time away from a busy home environment or because of the physical activity required to leave the house.

#### Prior Internet Experience

Generally, lack of confidence using computers hampered use of the intervention by many participants with little or no Internet experience. Participants with little Internet experience were likely to forget how to use the intervention and felt uncomfortable asking for help.

79-year-old male, basic Internet experience P0110: “I didn’t think I would use it a lot because… I get frustrated if the machine doesn’t immediately do what I want it to do and then I have to call my wife in and we have to sit there together.”

Participants were aware that family members and library or research staff could provide assistance, but felt embarrassed to reveal their lack of computer skills or that they had forgotten previous instructions.

53-year-old male, no previous Internet experience P0308: “You did volunteer to help me and I was embarrassed”

81-year-old female, no previous Internet experience P0320: “they were very helpful in the library I might say, but it was a little bit embarrassing admitting to your inadequacies”

Qualitative analysis also identified themes related to participant use of the intervention that add to, rather than explain the quantitative results. These included other themes related to participant need for information and support and their perceptions of the intervention.

The participants’ perceived need for help with CHD was related to more than the length of time since their diagnosis of CHD or cardiac event. Their perceived need was also related to their perceptions of CHD, to the inadequacy of existing sources of information and support, and to competing priorities. There was a strong connection between participants’ perceived need for help with CHD and their use of the web-based intervention.

#### Participant Levels of Need and Perceptions of their CHD

Many felt their CHD was not as severe as in other patients. This view was often based on whether or not they had experienced a heart attack.

79-year-old male, basic Internet experience P0110: “Well very fortunately none of the problems that other people have with heart problems. I haven’t, I didn’t have a heart attack, I had a bypass.”

Others judged the severity of their condition by whether they were currently experiencing any symptoms of CHD.

79-year-old male, experienced Internet user P0802: “… symptoms wise I do not have any heart problem… I had [a] heart attack… and so there’s obviously, its effect is there within me in some way, but it does not affect my daily life and I do not have any pain”

In addition, symptoms were often not perceived as problematic because they quickly resolved or were attributed to other causes (eg, other health condition, the weather, age).

#### Levels of Need and Adequacy of Existing Sources of Information and Support

Views on this differed greatly between participants and focused on the level of access to health professionals with sufficient time and expertise. Several participants felt they had good access to trusted health professionals and had no need to seek additional information.

66-year-old male, experienced Internet user P0608: “I’m not shy in coming forward… I ask him you know … always go to the specialist and that’s it. If I don’t get the right answer I go and ask another one…

Others had no desire to question the advice they received from health professionals.

79-year-old male, basic Internet experience P0110: “… why sort of double check something that somebody tells you… whom you trust… if your website or your answers would have been the same as ours well that confirms it, but I didn’t feel I was in need of confirmation.”

However, some participants felt that their health professionals had insufficient time to address their queries and concerns. For them, the intervention played an important role in dealing with this issue.

82-year-old male, experienced Internet user P0101: “… the cardiologist and GP, I only get very limited information from them. Mainly from the cardiologist but the amount of information he can give me in the time that he can devote to me is very limited and just… highlights points… which often I want to know more about”

#### Levels of Need and Competing Priorities

Intervention use was greatly affected by events in other areas of the participants’ lives. Those that felt little need for heart disease information and support were often busy with other priorities and had little time to use the intervention.

79-year-old male, basic Internet experience P0110: “My wife and I fortunately lead a very busy life and we travel quite a lot still and so there’s rarely a time when I sort of sit at my desk and say now what can I do …when I prioritize things I have to do, there isn’t a great deal of time left…”

For others, concurrent health problems were more of a concern than their heart disease, so those took priority. This was particularly true when participants experienced frequent symptoms from concurrent conditions or when those conditions required daily management.

#### Perceptions of the Intervention

Perceptions of the intervention varied greatly between participants. In general, participants that held positive views of the intervention used it, although some with a low need for information and support or low confidence in using computers, made little use of the intervention, despite viewing it positively. Perceptions were based on comparisons with other sources of information, advice, and support. In general, the intervention was favorably compared to other websites because it provided quicker access to relevant information.

82-year-old male, experienced Internet user P0101: “It was a quick source for the information whereas previously I had to go over other websites or publications to get the information. This helped to centralize that I can go to the CHESS site, it would lead me to other links.”

The intervention was also perceived as more relevant than newspapers because it provided more information and was easier to understand.

79-year-old female, experienced Internet user P0121: “…it was giving me information that I wouldn’t have had otherwise… you wouldn’t read those sort of things in the paper… probably the information wouldn’t be there… you get maybe a page of it in the paper, but just little bits…”

However, newspapers and books were preferred by participants that only wanted brief information or that had little confidence in using computers.

81-year-old female, no previous Internet experience P0320: “I suppose I just didn’t get the facility in using a computer that I would have liked, the way I could using books… which I’m very familiar with of course.

R: So by comparison it was

P0320: It was hard work…”

Some participants preferred the intervention to contact with health professionals because it was easier to access and without time constraints.

79-year-old female, no previous Internet experience P0121: “… it’s very difficult because if I want to ask my doctor a question… I have to go through the receptionist …and I might not speak to my own doctor, so the doctor I speak to doesn’t really know me, and I think that’s very off-putting. Whereas if I can go get what I want from upstairs with no problem at all… just switching the computer on, then that’s great… I’d much rather do that”

However, participants were most critical of the intervention when they compared it to seeking or receiving information and support during a one-on-one discussion. As a result, the intervention was perceived as more difficult, less personal, and less effective as a means of communication.

72-year-old male, no previous Internet experience P0906: “I would rather go out and meet somebody and talk to them like this because I think… you can’t convey a lot of that over a forum”

## Discussion

### Main Results

Despite an inclusive design, only a small proportion of eligible patients with CHD participated in the study (N=168, 10.6%). There was a greater proportion of participants that were younger and male compared to the general CHD population. Participants were predominantly Caucasian and had a higher level of education. Most had been previously diagnosed with CHD a number of years ago with no recent cardiac event or CHD complication. Most had home Internet access and prior Internet experience.

Statistical and qualitative analyses showed that time since the most recent CHD diagnosis or cardiac event, access to home Internet, and prior Internet experience were important factors in whether participants used the intervention. Qualitative data provided explanations for how and why these factors influenced use or lack of use of the intervention. A recent cardiac event or complication seemed to increase use of the intervention, due to an increased need of the participant for information and advice on CHD. However, this finding has to be interpreted within the context of few patients with a recent cardiac event or recently diagnosed with CHD choosing to participate in the study. Participants with no history of a recent cardiac event or complication reported little need for self-management support.

Other qualitative findings placed the effect of time since diagnosis with participant perceptions of their heart disease, the adequacy of existing sources of support, and competing priorities in determining need for self-management support and intervention use.

The convenience of accessing the intervention at home encouraged use, whereas lack of home Internet access was a barrier to intervention use. Participants with little or no Internet experience showed a lack of confidence in using computers in general and felt uncomfortable seeking help, even when it was available. Interview data also suggest that participant perception of the intervention, specifically when compared to other sources of information, advice, and support, interacted with their level of need and confidence with computers to influence their use of the intervention. Gender and level of education did not significantly predict level of intervention use. Older participants made greater use of the intervention compared to younger participants.

### Comparisons to Previous Studies

A low rate of participation and a high proportion of Caucasian well-educated patients mirror the problems found in generic self-management programs [7,8,10]. Contrary to these programs and patterns of internet use for health information [23,25], participants in this study were predominantly male. However, this has been shown to be common in secondary prevention interventions for CHD [6,9]. Gender bias in participation rates could be the result of the low appeal of the intervention or increased barriers to participation among women, rather than the high appeal of the intervention to men. Overall, these results suggest that the study was not successful in reaching individuals most in need. Moreover, participant clinical features and qualitative data suggest that participants’ CHD was relatively unproblematic.

Key factors in the digital divide (gender, age, and education) did not appear to affect participant level of use of the intervention. In fact, it was observed that older participants were more likely to make use of the intervention. This is a counterintuitive finding and should be interpreted with caution, since older participants were not well represented in the sample. Sample characteristics suggest that the older participants in this study might not be representative of older CHD patients in general. Qualitative findings did not provide a clear explanation for the effect of age on intervention use, although increased free time among retired participants might be a factor. In general, these findings support those of similar studies on the use of the Internet for obtaining health information [43,44], and suggest that, when participants are provided Internet access, disparities associated with the digital divide are likely to disappear.

However, ease of access to public Internet services did not encourage many of the CHD patients without home Internet access to participate in the study. Moreover, lack of home Internet access and prior Internet experience were significant predictors of lower use of the intervention. This appeared to be due to the inconvenience of public Internet access, lack of confidence with computers, and discomfort in asking for assistance. This suggests that factors other than ease of access or availability of public Internet services are required to overcome the digital divide. Barriers and aids to Internet use, beyond issues of access, have been explored in a recent small-scale study [45]. Investigators provided computer novices from low socioeconomic groups with free home computer systems, broadband Internet access, monthly computer training courses, and technical support for a year. Regular training and technical support, in addition to social support from other participants, facilitated general computer and Internet use beyond the availability of home Internet access [45]. However, the feasibility of such an approach on a larger scale outside the research setting remains an issue.

### Strengths and Limitations

The strengths of this study are its inclusive and mixed methods design. The study design included recruitment of participants from diverse socioeconomic and ethnic backgrounds that were offered a self-management intervention made available to them through public and home Internet access. Mixed quantitative and qualitative methods enabled the authors to both quantify and explain the factors influencing the use of the intervention. Another strength of the study was the web-based intervention used: it was designed by the experienced CHESS team and further developed to meet the particular needs of UK patients.

However, one limitation to the study was a lack of information about the large number of patients that were eligible to participate but chose not to. Access to this information was restricted for ethical reasons, based on their lack of consent. Comparison of participant data with general data from CHD registers and UK population surveys provides certain general conclusions about those that chose not to participate. However, the specific reasons behind their decision not to participate are unknown. Recruitment following a single written invitation to participate was ethically appropriate but might have played a role in the limited number of participants. The recruitment strategy might have been more successful in enrolling patients without home Internet or prior experience through the use of a more personal approach. Conclusions about the relationship between age, gender, level of education, date of recent cardiac event, CHD diagnosis or complication, and use of the intervention are limited by the lack of representation of these characteristics in the study sample.

### Conclusions

Despite an inclusive recruitment strategy, participants in this study seemed to have a higher level of education, better access to and experience of the Internet, and might have had fewer problems with their condition compared to that observed in the general CHD population. Predictors of use of the intervention by those who participated underlined participants’ relatively low need for information, advice, and support; the availability of home Internet access; and the level of experience using the Internet. This study suggests that availability of public Internet access is unlikely to be sufficient to help individuals overcome the digital divide. Equitable access to Internet services remains a significant challenge that could limit the potential of web-based interventions for overcoming health disparities through the use of self-management programs by chronically ill patients.
